# Nidogen 2 Overexpression Promotes Hepatosteatosis and Atherosclerosis

**DOI:** 10.3390/ijms252312782

**Published:** 2024-11-28

**Authors:** Ishita Kathuria, Aditi Prasad, Bal Krishan Sharma, Ravi Varma Aithabathula, Malvin Ofosu-Boateng, Maxwell A. Gyamfi, Jianxiong Jiang, Frank Park, Udai P. Singh, Bhupesh Singla

**Affiliations:** 1 Department of Pharmaceutical Sciences, College of Pharmacy, The University of Tennessee Health Science Center, Memphis, TN 38103, USA; ikathuri@uthsc.edu (I.K.); aprasad9@uthsc.edu (A.P.); raithaba@uthsc.edu (R.V.A.); mofosubo@uthsc.edu (M.O.-B.); mgyamfi@uthsc.edu (M.A.G.); jjiang18@uthsc.edu (J.J.); fpark@uthsc.edu (F.P.); usingh1@uthsc.edu (U.P.S.); 2 Cancer and Blood Diseases Institute, Cincinnati Children’s Hospital Medical Center, Cincinnati, OH 45229, USA; balkrishan.sharma@cchmc.org

**Keywords:** nidogen 2, hepatosteatosis, atherosclerosis, AMPK, NAFLD

## Abstract

Clinical and genetic studies strongly support a significant connection between nonalcoholic fatty liver disease (NAFLD) and atherosclerotic cardiovascular disease (ASCVD) and identify ASCVD as the primary cause of death in NAFLD patients. Understanding the molecular factors and mechanisms regulating these diseases is critical for developing novel therapies that target them simultaneously. Our preliminary immunoblotting experiments demonstrated elevated expression of nidogen 2 (NID2), a basement membrane glycoprotein, in human atherosclerotic vascular tissues and murine steatotic livers. Therefore, we investigated the role of NID2 in regulating hepatosteatosis and atherosclerosis utilizing Western diet-fed *Apoe*^−/−^ mice with/without *NID2* overexpression. Quantitative real-time PCR confirmed increased *NID2* mRNA expression in multiple organs (liver, heart, kidney, and adipose) of *NID2*-overexpressing mice. Male mice with *NID2* overexpression exhibited higher liver and epididymal white adipose tissue mass, increased hepatic lipid accumulation, and fibrosis. Additionally, these mice developed larger atherosclerotic lesions in the whole aortas and aortic roots, with increased necrotic core formation. Mechanistic studies showed reduced AMPK activation in the livers of *NID2*-overexpressing mice compared with controls, without any effects on hepatic inflammation. In conclusion, these findings suggest that NID2 plays a deleterious role in both hepatosteatosis and atherosclerosis, making it a potential therapeutic target for these conditions.

## 1. Introduction

The liver plays a central role in regulating systemic lipid metabolism and maintaining homeostasis. Disruptions in hepatic lipid metabolism lead to NAFLD development. NAFLD comprises a spectrum of pathological changes in the liver, which can be histologically classified into two categories: (a) nonalcoholic fatty liver (NAFL), characterized by lipid accumulation (steatosis) in more than 5% of hepatocytes without signs of hepatocyte injury, and (b) nonalcoholic steatohepatitis (NASH), defined as hepatic steatosis and inflammation, along with hepatocyte injury with or without fibrosis [1,2]. Over the past two decades, the global incidence of NAFLD has increased significantly from 25% to 32% [3], and this trend is expected to continue at an alarming rate. Nonetheless, until recently, there was no Federal Drug Administration (FDA)-approved pharmacological treatment for NAFLD or NASH [4]. In March 2024, the FDA approved Resmetirom, a liver-targeted agonist of thyroid hormone receptor β-selective for treating NASH. Regardless of its benefits, Resmetirom’s usage is associated with several side effects, including nausea, diarrhea, gallbladder-related side effects, and drug-induced liver toxicity. Moreover, it is contraindicated in patients with decompensated cirrhosis [5,6]. Given the high prevalence and complications associated with NAFLD, the development of additional novel therapeutic drugs with minimal side effects is essential. In addition, dysregulated hepatic lipid metabolism elevates circulating levels of low-density lipoprotein (LDL) particles, which induces vascular endothelium toxicity, promotes LDL infiltration into the arterial wall, and contributes to plaque formation, thereby linking NAFLD to atherosclerosis development [7]. Since NAFLD shares risk factors such as dyslipidemia, visceral obesity, and insulin resistance with ASCVD, patients with NAFLD are at higher risk for adverse ASCVD events. Notably, ASCVD is recognized as the major cause of death among NAFLD patients, independent of traditional risk factors [8]. However, our knowledge about the endogenous molecular factors and downstream signaling mechanisms responsible for dysregulated hepatic lipid metabolism and their roles in atherosclerotic lesion formation remains limited.

NID2 is a glycoprotein present in the basement membrane, where it interacts with collagen IV, perlecan, and collagen I to stabilize the membrane structure [9]. NID2 expression has been linked to the development of various cancers, including ovarian, lung, gastric, pancreatic, and oral squamous cell carcinoma [10,11,12,13,14]. Additionally, elevated *NID2* mRNA levels have been observed in human atherosclerotic echolucent calcified plaques [15,16], which was further confirmed by a study analyzing publicly available transcriptomic profiles of atherosclerotic arteries from the Gene Expression Omnibus database [17]. Interestingly, in murine models of vascular calcification and neointima formation, NID2 demonstrated a protective role against vascular calcification and helped in promoting the contractile phenotype of vascular smooth muscle cells (VSMCs), respectively [18,19]. This apparent contradiction suggests NID2’s diverse role in different cell types and disease models, highlighting the need for further studies. Nevertheless, its role in regulating hepatosteatosis and associated atherosclerosis has never been explored.

In this study, we investigated the role of NID2 in the pathogenesis of NAFLD and atherosclerosis by overexpressing *NID2* using an adeno-associated viral (AAV) vector in *Apoe*^−/−^ mice and Western diet feeding. We observed that NID2 protein expression is increased in both steatotic livers and atherosclerotic vascular tissues. *NID2* overexpression in male *Apoe*^−/−^ mice promoted hepatic steatosis, fibrosis, and atherosclerosis development. Interestingly, female mice with or without *NID2*-overexpression exhibited no differences in atherosclerosis development and hepatic fibrosis. Mechanistically, we found attenuated AMPK activation in the livers of *NID2*-overexpressing mice compared with controls, with no effects on hepatic inflammation. These findings provide the first experimental evidence of NID2’s detrimental role in the development of NAFLD and atherosclerosis.

## 2. Results

### 2.1. Expression of NID2 Protein Is Elevated in Human Atherosclerotic Arteries and Murine Steatotic Livers

**Figure 1:**
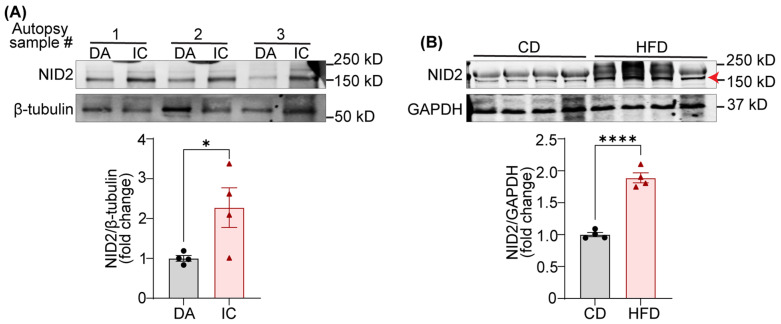
Expression of NID2 protein is elevated in human atherosclerotic arteries and murine steatotic livers. (**A**) Representative western blot images for NID2 and β-tubulin protein expression in human atherosclerotic inner curvature (IC) and non-atherosclerotic descending aorta (DA) vascular tissue. The bar diagram shows mean protein levels expressed as a ratio of NID2 to β-tubulin. (**B**) Representative Western blot images for NID2 (red arrowhead points to the correct band) and GAPDH in the livers of control diet (CD)- and calorie-matched high-fat diet (HFD, 12 weeks)-fed C57BL/6J mice. The bar diagram represents the mean NID2 protein expression (*n* = 4). Statistical analyses were performed using a two-tailed unpaired *t*-test (**A**,**B**). Data represent mean ± SEM. * *p* < 0.05, and **** *p* < 0.0001.

A recent RNA-sequencing study reported increased levels of *NID2* mRNA in human carotid artery echolucent plaques, which are lipid-rich lesions and associated with a higher risk of adverse cardiovascular events [16,20,21]. Additionally, a separate study utilizing liquid chromatography–mass spectrometry suggested an association between *NID2* mRNA expression and calcified hard plaques [15]. However, no previous studies have investigated the levels of NID2 protein in non-atherosclerotic and atherosclerotic arteries. Therefore, we performed immunoblotting experiments using tissue lysates from the atherosclerotic inner curvature (IC) of the aorta and non-atherosclerotic descending aorta (DA). The presence and absence of atherosclerosis was confirmed using Oil Red O (ORO) staining of frozen sections, as reported earlier [22]. The results demonstrated significantly elevated NID2 protein expression in atherosclerotic IC compared with non-atherosclerotic DA segments (Figure 1A).

Hepatosteatosis (deposition of lipids in the liver) is closely linked with the development of atherosclerosis [8,23]. However, no information is available on the expression of NID2 in steatotic livers. To investigate NID2 levels in steatotic livers, we conducted immunoblotting with liver tissue lysates from wild-type (C57BL/6) mice fed a control diet (CD) and calorie-matched high-fat diet (HFD). The hepatosteatosis in HFD-fed mice was confirmed with ORO staining. The immunoblotting data revealed increased hepatic expression of NID2 in HFD-fed mice compared with CD-fed mice, suggesting an association between hepatosteatosis and NID2 protein expression (Figure 1B). Similar results were obtained when NID2 expression in the livers of *Apoe*^−/−^ mice fed regular chow and a Western diet was compared (Supplementary Figure S1). Collectively, these data indicate that NID2 levels are augmented in both atherosclerotic arteries and steatotic livers.

### 2.2. NID2 Overexpression Enhances Liver and Epididymal White Adipose Tissue Mass in Male Mice

**Figure 2:**
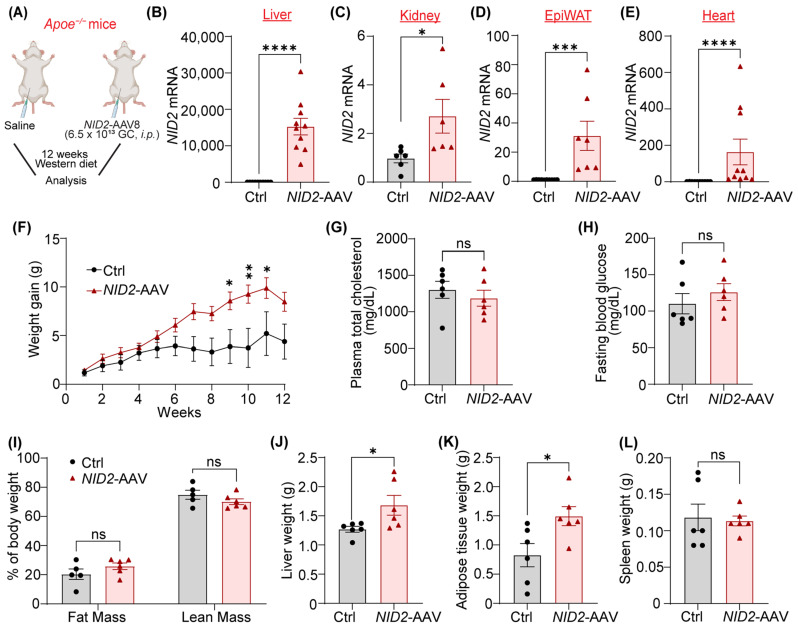
*NID2* overexpression enhances liver and epididymal white adipose tissue mass in male mice. (**A**) The schematic diagram illustrates the experimental plan. *Apoe*^−/−^ mice were injected with control (Ctrl) and *NID2*-AAV intraperitoneally, fed a Western diet for 12 weeks, and analyzed. (**B**–**E**) Male control and *NID2*-AAV-injected *Apoe*^−/−^ mice were utilized to measure *NID2* mRNA levels in various organs by qRT-PCR at least in duplicate. Bar diagrams represent mRNA expression in the liver (**B**, *n* = 10), kidney (**C**, *n* = 6), epididymal white adipose tissue (EpiWAT, **D**, *n* = 7–10), and heart (**E**, *n* = 10). Bar diagrams show body weight gain (**F**), plasma total cholesterol (**G**), fasting blood glucose (**H**), whole-body fat/lean mass (**I**), liver weight (**J**), adipose tissue weight (**K**), and spleen weight (**L**) (*n* = 5–6). A two-tailed unpaired *t*-test (**C**,**G**–**K**), two-tailed unpaired Mann–Whitney test (**B**,**D**,**E**,**L**), and two-way ANOVA followed by Sidak post hoc test for multiple comparisons (**F**) were utilized for statistical analyses. Data represent mean ± SEM. ns: non-significant. * *p* < 0.05, *** *p* < 0.001 and **** *p* < 0.0001.

Since our data shown in Figure 1 demonstrated increased NID2 levels in both atherosclerotic arteries and steatotic livers, we next aimed to examine the effects of *NID2* overexpression on the development of hepatosteatosis and atherosclerosis in mice. Therefore, we used male and female *Apoe*^−/−^ mice, a genetic model of both atherosclerosis and NAFLD [24,25]. These mice were injected with either control or *NID2*-AAV intraperitoneally and fed a Western diet for 12 weeks (Figure 2A). The quantitative real-time PCR (qRT-PCR) analysis showed overexpression of *NID2* in various organs, including liver, kidney, epididymal white adipose tissue, and heart of *NID2*-AAV-administered mice compared with control mice (Figure 2B–E). Throughout the 12-week Western diet feeding period, mice were weighed weekly, and no significant differences were observed in body weight between male and female control and *NID2*-overexpressing mice (Supplementary Figures S2 and S3A). However, weight gain was significantly higher in male *NID2*-AAV-injected mice compared with controls (Figure 2F). In addition, plasma total cholesterol, fasting blood glucose, whole-body fat, and lean mass were comparable between the two groups (Figure 2G–I and Supplementary Figure S3B–D). Interestingly, in male *NID2*-AAV-injected mice, mean liver weight (Figure 2J and Figure S4) and epididymal white adipose tissue (epiWAT, Figure 2K) weight were significantly higher in comparison to control mice. However, no such differences were observed in female mice (Supplementary Figure S3E,F). Moreover, spleen weights were not different between the two groups (Figure 2L and Figure S3G). These observations suggest that *NID2* overexpression in male mice increases liver and epididymal white adipose tissue weight but does not affect these parameters in female mice.

### 2.3. NID2 Overexpression in Mice Promotes Hepatic Lipid Accumulation and Fibrosis

**Figure 3:**
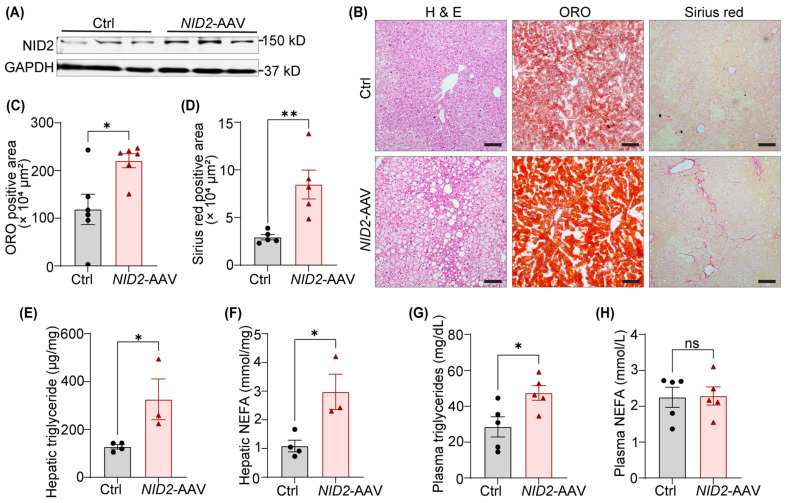
*NID2* overexpression in mice promotes hepatic lipid accumulation and fibrosis. Male *Apoe*^−/−^ mice were injected with control and *NID2*-AAV intraperitoneally, fed a Western diet for 12 weeks, and analyzed. (**A**) Representative Western blot images for NID2 and GAPDH protein expression in the livers of control and *NID2*-overexpressing mice (*n* = 3). (**B**) Representative images of liver sections stained with H & E (lipid droplets), ORO (neutral lipid accumulation), and Sirius red (fibrosis); scale bar 100 μm. (**C**–**H**) Bar diagrams represent lipid accumulation (**C**, *n* = 6), fibrosis area (**D**, *n* = 5), hepatic triglyceride (**E**, *n* = 3–4), NEFA levels (**F**, *n* = 3–4), plasma triglyceride (**G**, *n* = 5) and NEFA levels (**H**, *n* = 5), in control and *NID2*-AAV-injected mice. Statistical analyses were performed using a two-tailed unpaired *t*-test (**C**–**H**). Data represent mean ± SEM. ns: non-significant. * *p* < 0.05, and ** *p* < 0.01.

Since (a) NID2 expression is elevated in steatotic livers of HFD-fed mice, and (b) *NID2* overexpression increases liver weight in male mice, we investigated the role of NID2 in regulating hepatic steatosis and fibrosis. To begin, we confirmed hepatic NID2 expression in both control and *NID2*-AAV-injected mice using Western blotting. As expected, *NID2*-AAV administration significantly increased NID2 protein (Figure 3A) and mRNA levels (Figure 2B) compared with controls. Next, serial liver sections (paraffin/frozen) from similar regions of livers from male mice were stained with hematoxylin and eosin (H & E), ORO, and Sirius red to visualize lipid droplets, neutral lipid accumulation, and fibrosis, respectively (Figure 3B). These histological analyses demonstrated an increased frequency/size lipid droplets, enhanced lipid deposition, and higher hepatic fibrosis in male *NID2*-AAV-injected mice, in contrast with sex-matched controls (Figure 3B–D). Similar investigations were conducted on female liver samples. *NID2* overexpression significantly increased lipid droplet accumulation and elevated the ORO-positive area; however, no differences in liver fibrosis were found between the two female groups (Supplementary Figure S3H–J).

Hepatosteatosis is marked by the accumulation of triglycerides and the presence of free fatty acids in the liver [26]. Therefore, we next measured triglyceride and non-esterified fatty acid (NEFA) levels in hepatic samples of male mice. Our data showed a significant increase in the levels of hepatic triglyceride and NEFA in *NID2*-overexpressing mice compared with controls (Figure 3E,F). Moreover, plasma triglyceride levels were elevated in *NID2*-AAV-injected mice, while plasma NEFA levels were similar between the two groups (Figure 3G,H). Free fatty acids released by lipolysis in adipose tissues are diverted to the liver, suggesting a pivotal interaction between hepatic metabolism and adipose tissue in the pathogenesis of NAFLD [27]. Additionally, adipocyte hypertrophy has been linked with NAFLD development [28]; therefore, we performed H & E staining of epididymal white adipose tissue and found increased adipocyte size in male *NID2*-AAV-injected mice compared with the control group (Supplementary Figure S5). Altogether, these findings suggest that *NID2* overexpression contributes to hepatic lipid accumulation and fibrosis in male mice.

### 2.4. NID2 Overexpression Augments Atherosclerosis in Male Hypercholesterolemic Mice

**Figure 4:**
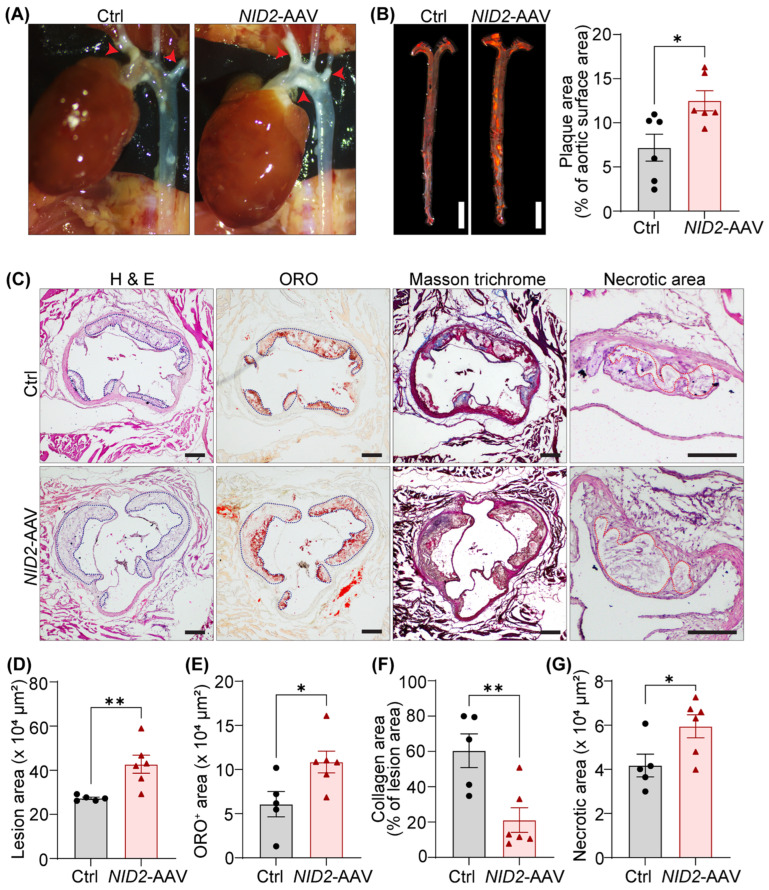
*NID2* overexpression augments atherosclerosis in male hypercholesterolemic mice. Male *Apoe*^−/−^ mice were injected with control (Ctrl) and *NID2*-AAV intraperitoneally, fed a Western diet for 12 weeks and analyzed. (**A**) Representative in situ images of the aortic arch (red arrowheads point to atherosclerotic lesions). (**B**) Representative ORO staining of whole aortas; scale bar 5 mm. The bar diagram represents ORO-positive areas in whole aortas (*n* = 6). (**C**) Representative images of aortic root cross-sections stained with H & E (lesion area and necrotic core), ORO (lipid accumulation), and Masson’s trichrome (collagen content); scale bar 200 μm. (**D**–**G**) Bar diagrams show lesion area (**D**), lipid deposition (**E**), collagen content (**F**), and necrotic core area (**G**) (*n* = 5–6). Statistical analyses were performed using a two-tailed unpaired *t*-test (**B**,**D**–**G**). Data represent mean ± SEM. * *p* < 0.05, and ** *p* < 0.01.

To explore the effects of dysregulated hepatic metabolism on the development of atherosclerosis, we investigated atherosclerotic lesion formation in control and *NID2*-overexpressing *Apoe*^−/−^ mice. In situ images of the aortic arch revealed a larger plaque area in *NID2*-AAV-injected mice compared to control mice (Figure 4A). *En face* ORO staining of whole aorta further confirmed a significant increase in atherosclerosis in male mice with *NID2* overexpression [% plaque area: 12.5 ± 1.14 (74.09% increase)] compared with controls (7.18 ± 1.53), indicating a detrimental role for NID2 in the development of atherosclerosis (Figure 4B). Interestingly, no differences in plaque burden were observed in female mice (Supplementary Figure S3K,L). Moreover, H & E staining performed on serial aortic cross-sections (4 sections/mouse, 90–100 µm apart) from male *NID2*-AAV-injected mice demonstrated a significantly higher atherosclerotic lesion area compared with sex-matched control tissue (42.71 ± 4.09 × 10^4^ µm^2^ vs. 27.29 ± 0.55 × 10^4^ µm^2^) (Figure 4C,D). Additional aortic root histological analyses showed increased ORO-positive area (lipid accumulation), reduced collagen (Masson trichrome), and enlarged necrotic area (acellular areas) in *NID2*-overexpressing mice compared with control animals (Figure 4C,E–G). Collectively, these data show that *NID2* overexpression in male hypercholesterolemic mice exacerbates atherosclerotic lesion formation, promotes necrotic core formation, and reduces plaque stability. In contrast, consistent with *en face* ORO staining results, aortic root histology revealed no significant differences in lesion area in female mice (Supplementary Figure S3M).

### 2.5. NID2 Overexpression Inhibits the Activation of Lipid Metabolism-Related Protein AMPK

**Figure 5:**
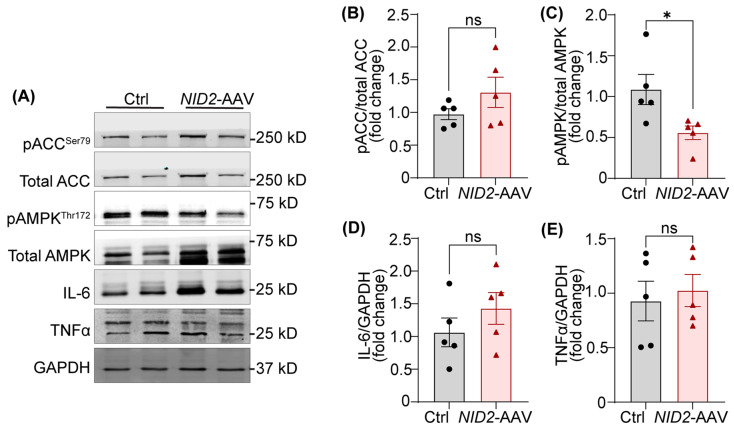
*NID2* overexpression inhibits the activation of the lipid metabolism-related protein AMPK. (**A**) Representative Western blot images for lipid metabolism and pro-inflammatory proteins utilizing liver lysates from control and *NID2*-AAV-injected mice. Bar diagrams represent mean protein expression (**B**,**C**) as the ratios of phospho-total proteins ACC (**B**) and AMPK (**C**), and protein levels of IL-6 (**D**) and TNFα (**E**) (*n* = 5). Statistical analyses were performed using a two-tailed unpaired *t*-test. Data represent mean ± SEM. ns: non-significant. * *p* < 0.05.

Our experiments as shown above revealed that *NID2* overexpression leads to increased hepatosteatosis and atherosclerosis. To uncover the underlying molecular mechanisms by which NID2 regulates hepatic lipid metabolism, we determined the expression of various proteins and genes involved in lipid metabolism and inflammation. Adenosine monophosphate-activated protein kinase (AMPK) serves as a key energy sensor, and its activation by phosphorylation promotes the metabolic switch from an anabolic to a catabolic state, shutting down ATP-dependent synthetic pathways to restore energy balance [29]. When activated, AMPK deactivates acetyl-COA carboxylase (ACC) via phosphorylation at serine 79, reducing the levels of malonyl-CoA, which is a precursor of fatty acid synthesis and an inhibitor of mitochondria fatty acid uptake via carnitine palmitoyltransferase-1 (CPT1) [30,31]. Therefore, we investigated the activation status of AMPK and ACC in liver tissue lysates from control and *NID2*-AAV-injected mice. Western blot analysis demonstrated decreased phosphorylation of AMPK (Thr 172) in *NID2*-overexpressing livers in comparison to control livers (Figure 5A,C). However, there were no significant differences in ACC phosphorylation between the two groups (Figure 5A,B). These results indicate that NID2 may regulate hepatic lipid metabolism by modulating AMPK activation without directly affecting ACC activity.

Inflammation plays a key role in promoting hepatocyte death and tissue injury in NAFLD [32]. To explore this, we performed qRT-PCR to determine the hepatic mRNA levels of various inflammatory genes including nitric oxide synthase 2 (*Nos2*), tumor necrosis factor alpha (*Tnfa*), and interleukin 6 (*Il6*). However, no significant differences were observed between the groups (Supplementary Figure S6). Consistently, Western blot data exhibited comparable TNFα and IL-6 levels in the livers of control and *NID2*-AAV-injected mice (Figure 5A,D,E). We also investigated the mRNA expression of genes involved in lipid uptake (*Msr1* and *Cd36*), fatty acid synthesis (*Fasn*), fatty acid transport (*Cpt1a*), and fatty acid beta-oxidation (*Ppara*); however, no significant differences were noted. Interestingly, *Ldlr* expression was significantly increased in mice injected with *NID2*-AAV, in contrast to controls (Supplementary Figure S6). Taken together, these results indicate that *NID2* overexpression in male mice may disrupt hepatic lipid metabolism by inhibiting AMPK activation.

## 3. Discussion

The liver plays a key role in systemic lipid metabolism by regulating the synthesis, uptake, storage, and efflux of cholesterol [8,33]. The prevalent liver disease NAFLD and its inflammatory form NASH are associated with the development of coronary artery calcification, atherosclerotic plaques, and increased carotid intima-media thickness [34,35]. These associations have stimulated interest in identifying the shared molecular mechanisms which drive these liver and vascular diseases. Nevertheless, our knowledge about the intrinsic molecular factors and downstream mechanisms responsible for dysregulated hepatic lipid metabolism and their roles in atherosclerotic lesion formation remains limited. Herein, we investigated the role of NID2 in regulating hepatosteatosis, fibrosis, and atherosclerotic lesion formation. Our results demonstrated (a) elevated NID2 levels in murine steatotic livers and human atherosclerotic vascular tissues, (b) increased hepatic steatosis and fibrosis in *NID2*-overexpressing mice, (c) exacerbated atherosclerosis in mice with *NID2* overexpression, and (d) reduced AMPK activation in the livers of *NID2*-overexpressing mice. Collectively, these findings suggest that NID2 contributes to the progression of both hepatosteatosis and atherosclerosis.

NID2 is a secretory glycoprotein, which is ubiquitously present in the basement membrane (BM) and helps to maintain BM stability [9,36]. Depending on the cell type and disease, NID2 plays both beneficial and detrimental roles. Previous studies have associated NID2 expression with various cancers, including gastric, ovarian, bladder, pancreatic, and esophageal squamous cell carcinoma [11,12,14,37]. Additionally, recent studies have linked *NID2* mRNA levels with human echolucent calcified plaques [15,16]. Further murine studies have identified the role of NID2 in non-atherosclerotic and atherosclerotic vascular calcification [18,38]. Interestingly, Chen et al. recently demonstrated a protective role of NID2 against aortic calcification induced by 5/6 nephrectomy, cholecalciferol-overload, and CaCl_2_ administration [18]. However, its role in regulating hepatosteatosis and atherosclerosis remains unknown. To address this gap, firstly, we investigated the expression of NID2 protein in human atherosclerotic arterial tissues and murine steatotic livers. Consistent with upregulated *NID2* expression in non-atherosclerotic and atherosclerotic calcified vascular tissues, increased NID2 protein expression was observed in atherosclerotic arteries and steatotic livers [15,16]. To explore NID2’s role in these diseases, we induced AAV-mediated overexpression of the *NID2* gene in *Apoe*-deficient mice. AAVs are widely used tools in research for their ability to efficiently transduce various cell types, allow long-term gene expression, and avoid integration into the host genome, reducing the risk of mutagenesis. Furthermore, this approach facilitates the rapid generation of mouse models without the need of breeding for several generations.

One of our notable observations was the significant increase in liver and EpiWAT weight in male mice overexpressing *NID2* compared to controls. This increase in liver weight, a hallmark of hepatosteatosis [39], indirectly suggests that NID2 may exacerbate hepatic fat accumulation in males following Western diet feeding. The lack of difference in plasma cholesterol, fasting glucose, and body fat composition between the groups indicates that NID2’s effects on liver and epiWAT mass are independent of systemic metabolic changes, such as overall cholesterol or glucose regulation. Interestingly, female mice did not exhibit any differences in liver or adipose tissue weights despite *NID2* overexpression, which points to a protective sex hormone/chromosome-regulated mechanism in females or a differential response to *NID2* overexpression. Future studies are warranted to determine the mechanisms of observed sex-specific responses.

Our data demonstrate that *NID2* overexpression in male mice promotes hepatic steatosis, as evidenced by increased triglyceride, NEFA levels, and ORO staining of liver frozen sections, as well as increased plasma triglycerides. These findings are consistent with previous studies that implicate lipid dysregulation as a characteristic of NAFLD [40,41]. While plasma NEFA levels did not differ between control and *NID2*-overexpressing mice, the elevated hepatic NEFA levels suggest that NID2 may alter the balance between hepatic lipid uptake and secretion. Free fatty acids, primarily derived from lipolysis in adipose tissue, are known to be transported to the liver, where they contribute to the pathogenesis of NAFLD [42]. In this context, the increased adipocyte size observed in *NID2*-overexpressing male mice further supports the link between adipose tissue dysfunction and hepatic lipid accumulation, a well-established mechanism in NAFLD [27]. Additionally, in male mice, overexpression of *NID2* increased liver fibrosis, which is a key determinant of the progression of simple steatosis to NASH [43]. These findings align with a previous report suggesting a connection between hepatic lipid accumulation and fibrosis in NAFLD [44]. Interestingly, though female mice exhibited increased hepatic lipid accumulation following *NID2* overexpression, no significant differences in fibrosis were noted. These sex-specific differences in fibrosis have also been observed in other models of liver disease and may be influenced by hormonal factors or differences in liver metabolism between males and females [45]. It is known that NAFLD prevalence and severity are higher in men in comparison to women during the reproductive age. However, after menopause, women develop NAFLD at a faster rate, suggesting a protective role of female sex hormones such as estrogen against NAFLD. Animal models also tend to follow a similar trend with higher severity and occurrence of hepatic steatosis, and pro-fibrotic/inflammatory cytokines in males than females, reinstating the differences we observed in our study [45,46].

The development of atherosclerosis is regulated by both systemic metabolic factors and local cellular responses [47]. In line with our hepatic lipid metabolism studies, *NID2* overexpression aggravated aortic atherosclerosis progression in male mice, suggesting a pivotal role of NID2 in atherogenesis. Further, *NID2*-overexpression led to increased lesion size, enhanced lipid accumulation, reduced collagen content, and enlarged necrotic cores in aortic root sections. These data are in compliance with the upregulated expression of *NID2* in atherosclerotic calcified vascular tissues, indicating the deleterious role of NID2 in vascular diseases [15,16]. In contrast, a recent study reported the protective role of NID2 in vascular calcification [18]. The authors observed attenuated NID2 levels in murine calcified aortas and calcified primary rat VSMCs, and showed suppression of vascular calcification in global *Nid2*^−/−^ mice [18]. Another study from the same research group highlighted NID2’s involvement in promoting the contractile phenotype of VSMCs [19]. Both of these studies emphasized the beneficial role of NID2 in regulating the VSMC phenotype. Arteries are composed of different layers with various cell types, including endothelial cells, VSMCs, adventitial fibroblasts, lymphatic endothelial cells, etc. However, the specific role of NID2 in different vascular cell types in the context of vascular pathologies remains to be investigated utilizing cell-specific *NID2*-deficient mice. It is possible that NID2 is playing different roles in different vascular cells. Furthermore, NID2 is recognized as an endogenous ligand for leucine-rich repeat-containing G protein-coupled receptor 4 (LGR4) [18]. Future studies are warranted to discover the other potential receptors of NID2 and investigate atherosclerosis in mice with cell-specific *Lgr4* deletion combined with NID2 overexpression to clarify the role of the NID2-LGR4 axis in atherogenesis. Additionally, reduced hepatic cholesterol efflux capacity in individuals with NAFLD has been linked to the presence of subclinical atherosclerosis [48], hinting that NID2 overexpression in hepatocytes may suppress cholesterol efflux, thereby leading to increased atherosclerosis. Comparable atherosclerosis in female control and *NID2*-overexpressing mice suggests sex-specific responses and implies that factors such as hormonal differences or sex-specific gene regulation modulate the effects of NID2 in atherosclerosis. For instance, estrogen has been shown to exert protective effects against atherosclerosis by modulating lipid metabolism and reducing inflammation [49,50]. Therefore, it is possible that sex hormones may be counteracting pro-atherogenic effects of NID2 in females.

To investigate the molecular mechanisms by which NID2 regulates hepatosteatosis and atherosclerosis, we examined the expression of various proteins and genes involved lipid metabolism. AMPK is a master switch in hepatic metabolism and its activation via phosphorylation (Thr 172) reduces hepatosteatosis by promoting fatty acid oxidation and inhibiting lipid production in the liver [51,52,53]. Phosphorylated AMPK inactivates ACC (Ser 79 phosphorylation), leading to increased fatty acid oxidation and reduced fatty acid synthesis [30,31]. Our data demonstrated reduced AMPK activation in the livers with *NID2* overexpression, with no changes in the activation status of ACC, hinting an ACC-independent function of AMPK in hepatosteatosis. These findings are consistent with a study by Zordoky et al., which also showed ACC-independent effects of AMPK in myocardial fatty acid oxidation [54]. However, it is unknown how NID2 regulates AMPK phosphorylation. It is possible that NID2, via inhibiting protein kinase C activation, suppresses AMPK activation [18]. Other AMPK-regulated factors such as the liver X receptor and sterol regulatory element-binding protein 1c (SREBP1c) may be mediating pro-steatotic/atherogenic effects of NID2 in mice [55]. We observed no significant differences in hepatic mRNA levels of various lipid uptake, fatty acid synthesis, fatty acid transport, and fatty acid beta oxidation genes, except *Ldlr* between control and *NID2*-AAV-injected mice. *Ldlr* mRNA expression was elevated in mice with *NID2* overexpression, which may represent a compensatory mechanism to clear excess lipids from circulation. Another possibility is that *NID2* overexpression increases proprotein convertase subtilisin/kexin type 9 levels, leading to degradation of LDLR protein and its reduced expression on the surface of hepatocytes, despite elevated mRNA levels [56], potentially contributing to the development of hepatosteatosis in these mice.

A major limitation of the present study is the utilization of *NID2*-AAV under the control of the ubiquitous cytomegalovirus promoter, which drives gene expression in multiple organs in mice. To better understand the cell-specific effects of *NID2* overexpression in NAFLD and atherosclerosis, future investigations with *NID2*-AAV with cell-specific promoter (hepatocytes/vascular cells) are required. Further, in vivo studies using cell-specific *NID2* deficiency are needed to investigate the precise role of endogenous NID2 in the pathophysiology of these diseases. Additionally, further investigations into the sex-specific roles of NID2 are warranted, including the use of ovariectomized female mice or the treatment of sex hormones, such as estradiol (E2), in male mice.

In conclusion, the present study, for the first time, demonstrates the detrimental role of NID2 in hepatosteatosis and atherosclerosis. The presented results suggest that blocking NID2-induced signaling may serve as potential therapeutic approach to suppress both NAFLD and atherosclerosis.

## 4. Materials and Methods

### 4.1. Animals and AAV Production

Eight- to ten-week-old male and female *Apoe*^−/−^ mice (The Jackson Laboratory, Bar Harbor, ME, USA, stock # 002052) were used in the present study. One group of *Apoe*^−/−^ mice, designated as control, received an intraperitoneal injection of saline, while another group was administered a single dose of a recombinant AAV expressing human *NID2* gene (AAV8-h*NID2*, referred to as *NID2*-AAV, 6.5 × 10^13^ viral genomes, IP) under the ubiquitous cytomegalovirus promoter, to induce *NID2* overexpression. The plasmid DNA for the *NID2* vector construct (pAAV-h*NID2*-CMV-amp, # 31829101) was obtained from Applied Biological Materials Inc., Richmond, Canada. The plasmid was packaged into AAV serotype 8 capsid and purified using a density gradient iodixanol solution at the Cincinnati Children’s Hospital Medical Center Viral Vector Core [RRID:SCR_022641)-VVL]. Eight- to ten-week-old male wild-type C57BL/6J mice were fed a control diet (Research Diets, Inc., New Brunswick, NJ, USA, D12450J) or a calorie-matched high-fat diet (Research Diets, Inc., New Brunswick, NJ, USA, D12492) for 12 weeks to determine NID2 protein levels using immunoblotting. All mice were housed in a climate-controlled vivarium (12-h light/dark cycle). All animal experiments were conducted according to the National Institutes of Health Guide for the Care and Use of Laboratory Animals, and performed after obtaining approval from the Institutional Animal Care and Use Committee of the University of Tennessee Health Science Center at Memphis, TN, USA (# 22-0319, dated 18 January 2022).

### 4.2. Atherosclerotic Lesion Analysis

Control and *NID2*-AAV-injected *Apoe*^−/−^ mice were fed a Western diet (Inotiv, Indianapolis, IN, USA, #TD.88137) for 12 weeks to induce hypercholesterolemia and atherosclerosis. In the twelfth week of feeding, whole-body fat and lean mass were measured using the EchoMRI Body Composition Analyzer. Fasting blood glucose (ReliOn Prime Blood Glucose Monitoring System, Bentonville, AR, USA) was determined following a 16 h fast, just before euthanasia. Mice were anesthetized by isoflurane inhalation (3%), and blood (via cardiac puncture), heart, aorta, epididymal adipose tissue, and other tissues were collected for further analysis. Similar regions of the liver from each mouse were processed for histochemistry and molecular analysis. Plasma total cholesterol was determined utilizing the Amplex Red cholesterol assay (Molecular Probes, Eugene, OR, USA, A12216). In situ images of the abdominal area, aortic arch, and heart were captured using a Leica S6E stereomicroscope fitted with a camera.

To assess the atherosclerotic lesion burden in whole aortas, *en face* ORO staining was performed after fixing aortas in 4% paraformaldehyde (PFA). A 2% ORO solution (ThermoFisher Scientific, Ward Hill, MA, USA, A12989.22) was used for staining. Aortas were opened longitudinally, and images were captured to quantify ORO-positive areas. To determine the lipid deposition in the aortic roots, the upper halves of fixed hearts were embedded in optimal cutting temperature (OCT) compound (Fisher Healthcare, Houston, TX, USA, 23-730-571), and serial frozen cross-sections (7 μm) were stained with 2% ORO solution. Images were captured using an Olympus BX43 inverted microscope. Four sections per mouse, spaced 90–100 μm apart, were stained and analyzed, and the mean area of the four sections reported. ORO-positive areas were quantified using Image Pro plus software (Media Cybernetics, Bethesda, MD, USA, version 7.0).

### 4.3. Hepatic Lipid Accumulation, Triglyceride, and Non-Esterified Fatty Acid Quantitation

To investigate the lipid accumulation in the liver, a similar region of the PFA-fixed and sucrose-dehydrated liver from each mouse was embedded in an OCT compound, and cryo-sectioning was performed. Frozen liver sections (7 μm) were stained with 2% ORO for 10 min at room temperature and counterstained with hematoxylin (Fisher Healthcare, Houston, TX, USA, 22-220-100). For each mouse, at least two sections were stained, and images of five to six random microscopic fields were captured. Image Pro Plus software was used for image analysis.

Liver homogenates were used to extract total lipids using the methanol and chloroform method. Plasma/hepatic triglyceride levels and NEFA levels were quantified following the standard protocols (Wako Pure Chemical Industries, Richmond, VA, USA, 992-02892, 992-02892, 464-01601, 995-34791, and 999-34691), as described previously [57].

### 4.4. Histochemistry

At least four aortic root sections per mouse, spaced 90–100 μm apart, were stained and analyzed, and the mean area of four sections was reported. Frozen serial cross-sections of aortic roots were washed twice with phosphate-buffered saline (PBS) and stained with H & E (Fisher Healthcare, Houston, TX, USA, 22-220-100 and 22-220-104) as described previously [22,58,59] to evaluate the total lesion and necrotic area. Masson’s trichrome staining (Richard Allan Scientific LLC, Kalamazoo, MI, USA, 22-110-648) was performed following the standard protocol to analyze the collagen content.

Frozen or paraffin liver sections were stained with H & E and Sirius red (Fisher Healthcare, Houston, TX, USA, 26357-02) as per the manufacturer’s instructions. H & E staining of liver sections was used to visualize the lipid globules, while Sirius red staining was employed to analyze the collagen content (fibrosis). A mean of five to six random microscopic fields per mouse was reported. All image analyses were performed utilizing Image-Pro Plus software.

### 4.5. Western Blotting

Murine liver and human vascular tissue samples were homogenized in radio-immunoprecipitation assay lysis buffer (RIPA, ThermoScientific, Ward Hill, MA, USA #89900) supplemented with protease and phosphatase inhibitor cocktail (ThermoScientific, Ward Hill, MA, USA #A32959). Equal amounts of protein were separated on SDS-PAGE gels, and resolved bands were transferred onto nitrocellulose membranes (Li-Cor Biosciences, Lincoln, NE, USA). Membranes were blocked (Intercept blocking buffer, Li-Cor Biosciences, Lincoln, NE, USA, #927-60001) and incubated overnight at 4 °C with indicated primary antibodies. The next day, membranes were washed and probed with IRDye-conjugated secondary antibodies (Li-Cor Biosciences, Lincoln, NE, USA). After washing, membranes were scanned with an Odyssey DLx Infrared Imaging System (Li-Cor Biosciences, Lincoln, NE, USA), and band intensities were determined using the NIH ImageJ software (version 1.53a). The following primary antibodies were used: NID2 (Proteintech, Rosemont, IL, USA #13530-1-AP), total ACC (Cell Signaling Technology, Danvers, MA, USA, #3662S), pACC^Ser79^ (Cell Signaling Technology, Danvers, MA, USA, #11818S), total AMPKα (Cell Signaling Technology, Danvers, MA, USA, #2793S), pAMPKα^Thr172^ (Cell Signaling Technology, Danvers, MA, USA, #2535S), IL-6 (Cell Signaling Technology, Danvers, MA, USA, #12912S), TNFα (Cell Signaling Technology, Danvers, MA, USA, #11948T), β-Tubulin (Cell Signaling Technology, Danvers, MA, USA, #86298S), and GAPDH (Santa Cruz Biotechnology, Dallas, TX, USA #sc-365062). All the antibodies except GAPDH and β-Tubulin were used at a 1:1000 dilution. GAPDH and β-Tubulin were utilized at a 1:2000 dilution.

### 4.6. Quantitative Reverse-Transcriptase PCR

Liver tissue samples were homogenized in TRIzol reagent (ThermoFisher Scientific, Ward Hill, MA, USA, #15596018), and total RNA was extracted according to the manufacturer’s instructions. Complementary DNA was synthesized using the RevertAid RT Reverse Transcription Kit (ThermoFisher Scientific, Ward Hill, MA, USA, #K1691). The qRT-PCR was performed using PowerUp SYBR Green Master Mix (ThermoFisher Scientific, Ward Hill, MA, USA, #A25742) in a QuantStudio 3 Real-Time PCR System (Applied Biosystems, Waltham, MA, USA). Relative gene expression was calculated using the 2^−ΔΔCt^ method and *Gapdh* as a housekeeping gene. Primer sequences used for qRT-PCR are listed in Supplementary Table S1.

### 4.7. Statistical Analysis

Sample sizes (*n*) for each experiment are mentioned in the figure legends. Statistical analyses were conducted using GraphPad Prism 10 (La Jolla, CA, USA). The normality of the data was assessed by the Shapiro–Wilk test. Comparisons between the two groups were performed using a two-tailed Student’s *t*-test for parametric data or a Mann–Whitney U test for non-parametric data. For parametric tests, the same standard deviation was assumed across groups. A *p*-value < 0.05 was considered statistically significant.

## Data Availability

The data presented in this study are available upon reasonable request from the corresponding author.

## Supplementary Materials

The following supporting information can be downloaded at: https://www.mdpi.com/article/10.3390/ijms252312782/s1.
