# Supplementary material for: Nidogen 2 Overexpression Promotes Hepatosteatosis and Atherosclerosis
Source: Int J Mol Sci. 2024 Nov 28;25(23):12782. doi: 10.3390/ijms252312782 (PMC11641205; doi:10.3390/ijms252312782)
Supplement: Supplementary file 1 [file ijms-25-12782-s001.zip › ijms-3307506-supplementary.pdf]

Supplementary Materials

Table S1: List of primers used for mRNA quantitation using quantitative real-time PCR.

**Gene name**	**Primer sequences**
Human *NID2*	F: 5'-TAG GCG CTT ACG AGG AGG TCA A-3'
	R: 5'-TAT CAG ACC CAT CAG ATG CCA AAA C-3'
Mouse *Nos2*	F: 5'-GTT CTC AGC CCA ACA ATA CAA GA-3'
	R: 5'-GTG GAC GGG TCG ATG TCA C-3'
Mouse *Tnfa*	F: 5'-TCC CAG GTT CTC TTC AAG GGA-3'
	R: 5'-GGT GAG GAG CAC GTA GTC GG-3'
Mouse *Ldlr*	F: 5'-ACC TGC CGA CCT GAT GAA TTC-3'
	R: 5'-GCA GTC ATG TTC ACG GTC ACA-3'
Mouse *Il6*	F: 5'-CAC AAG TCG GAG GCT TAA T-3'
	R: 5'-GTG CAT CAT CGT TCG TCA TAC-3'
Mouse *Cd36*	F: 5'-ATG GGC TGT GAT CGG AAC TG-3'
	R: 5'-TTT GCC ACG TCA TCT GGG TTT-3'
Mouse *Msr1*	F: 5'-CTG GAC TGA CGA AAT CAA GGA A-3'
	R: 5'-TGG AGG AGA GAA TCG AAA GCA-3'
Mouse *Fasn*	F: 5'-TTC CAA GAC GAA AAT GAT GC-3'
	R: 5'-AAT TGT GGG ATC AGG AGA GC-3'
Mouse *Cpt1a*	F: 5'-ACC ACT GGC CGC ATG TCA AG-3'
	R: 5'-CAG CGA GTA GCG CAT AGT CA-3'
Mouse *Gapdh*	F: 5'-AGG TCG GTG TGA ACG GAT TTG-3'
	R: 5'-GGG GTC GTT GAT GGC AAC A-3'

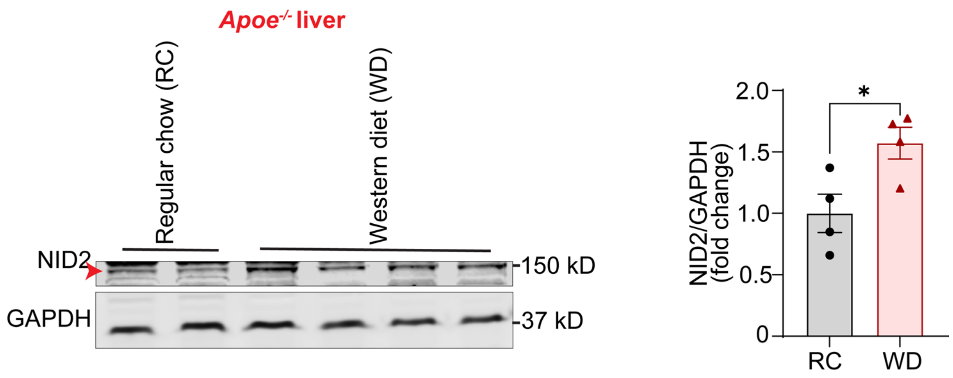

Figure 1. Expression of NID2 protein is increased in the livers of Western diet-fed *Apoe*$^{-/-}$ mice. Representative western blot images for NID2 (marked by red arrowhead) and GAPDH expression in the livers of regular chow (RC)- and Western diet (WD, Inotiv, TD88137, 12 weeks)-fed *Apoe*$^{-/-}$ mice. The bar diagram represents the mean NID2 protein expression (n = 4). Statistical analyses were performed using a two-tailed unpaired t-test. Data represents means ± SEM. *$P < 0.05$.

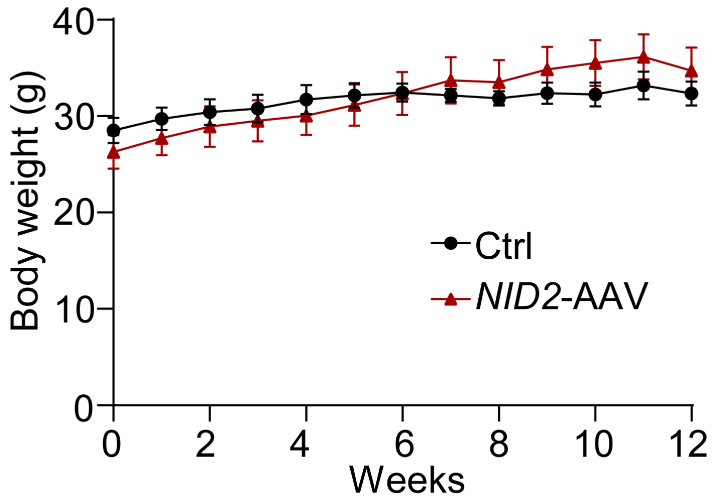

Figure S2: *NID2* overexpression in male *Apoe*$^{-/-}$ mice does not affect overall weight. Eight- to ten-week-old male *Apoe*$^{-/-}$ mice were injected with control (Ctrl) and *NID2*-AAV intraperitoneally, fed a Western diet for 12 weeks, and analyzed. Bar diagrams show the mean body weight. Statistical analyses were performed using a two-way ANOVA followed by Sidak post hoc test for multiple comparisons. Data represent mean ± SEM.

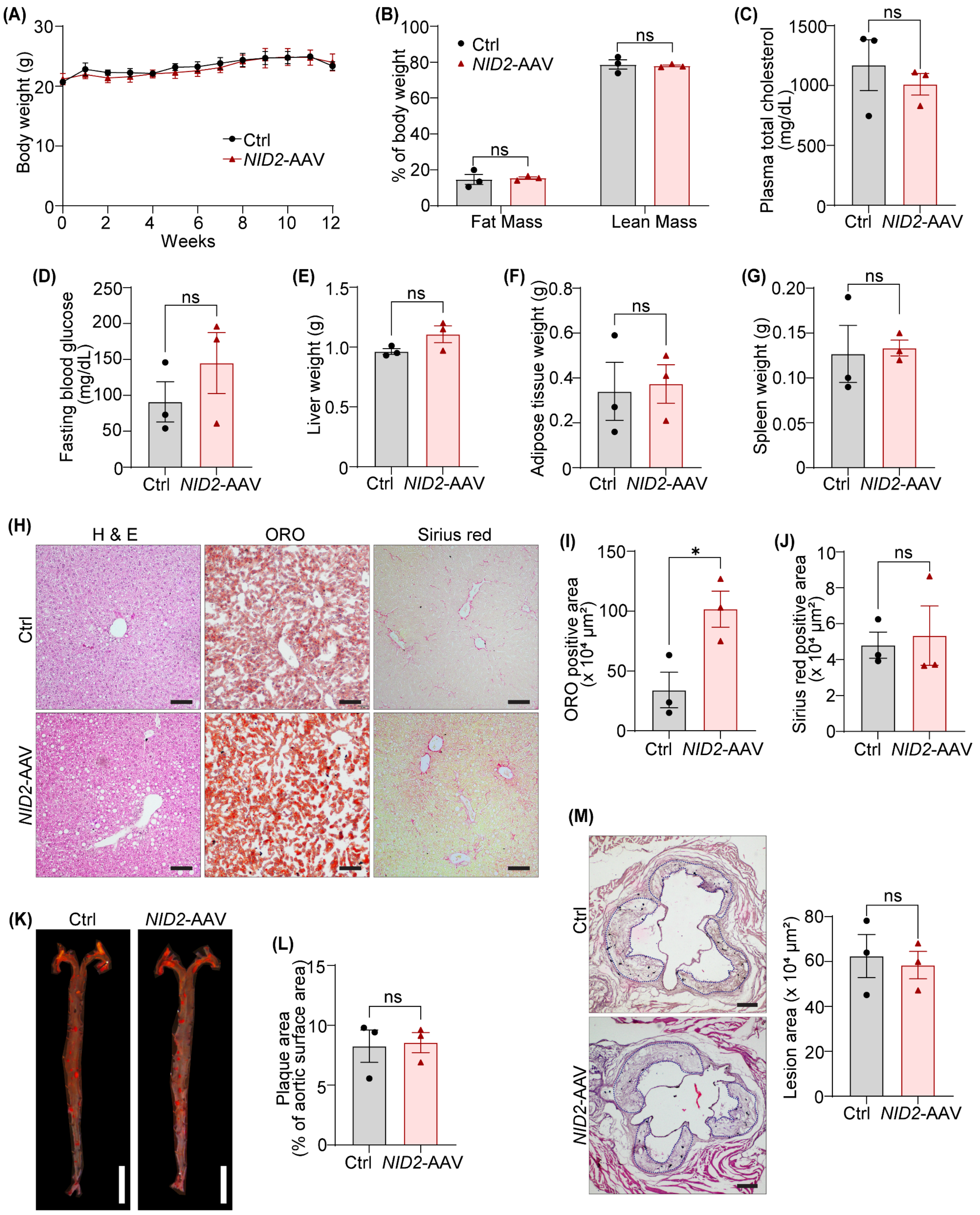

Figure S3: *NID2* overexpression in females elevates hepatic lipid accumulation. Female *Apoe*$^{-/-}$ mice were injected with control and *NID2*-AAV intraperitoneally, fed a

Western diet for 12 weeks, and analyzed. **(A - G)** Bar diagrams represent body weight **(A)**, whole-body fat mass and lean mass **(B)**, plasma total cholesterol **(C)**, fasting blood glucose **(D)**, liver weight **(E)**, adipose tissue weight **(F)**, and spleen weight **(G)**. **(H)** Representative images of liver sections stained with H & E (lipid droplets), ORO (neutral lipid accumulation) and, Sirius red (fibrosis), scale bar 100 µm. Bar diagrams represent lipid accumulation **(I)** and fibrosis area **(J)** in female mice. **(K)** Representative *en face* ORO staining of whole aortas, scale bar 5 mm. **(L)** Bar diagram represents ORO-positive areas in whole aortas. **(M)** Representative images of aortic root cross-sections stained with H & E, scale bar: 200 µm. Bar diagram represents lesion area, ($n = 3$). Statistical analyses were performed using a two-way ANOVA followed by Sidak post hoc test for multiple comparisons **(A)**, and a two-tailed unpaired t-test (**B - G, I, J, L and M)**. Data represent mean ± SEM. $^*P < 0.05$.

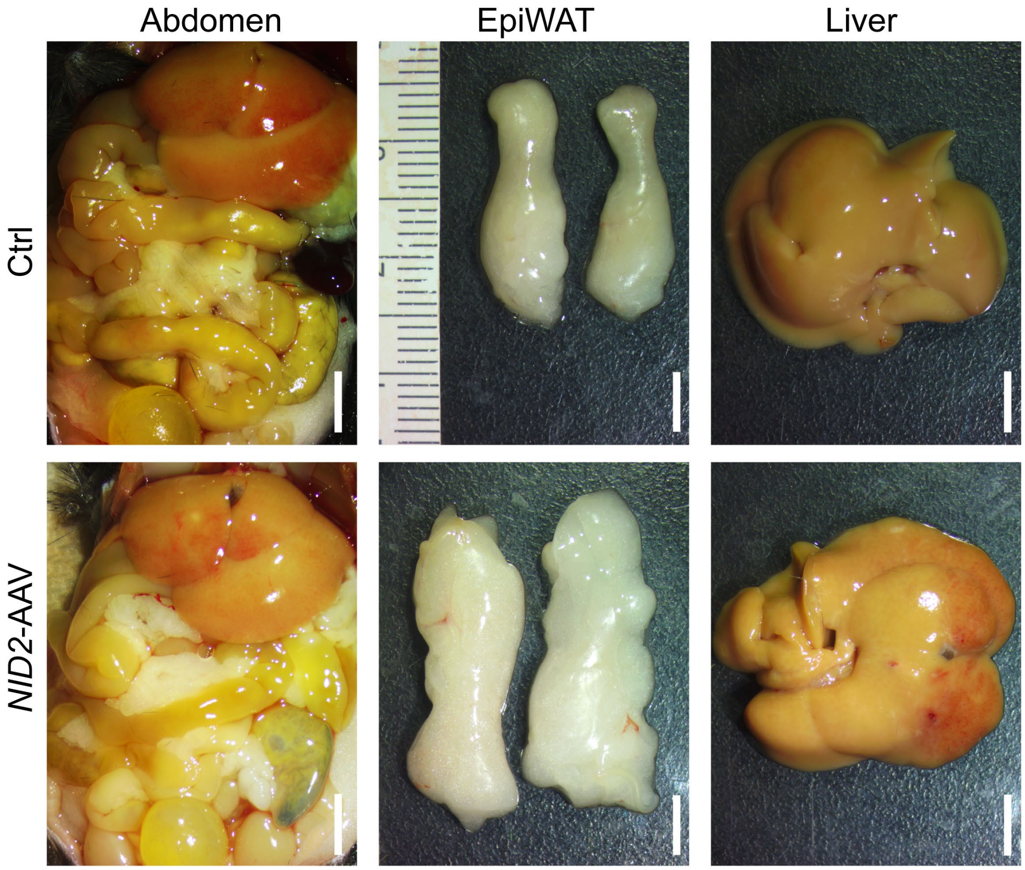

Figure S4: Representative *in situ* images of the abdomen, epididymal white adipose tissue and liver of control and *NID2*-AAV-injected male mice.

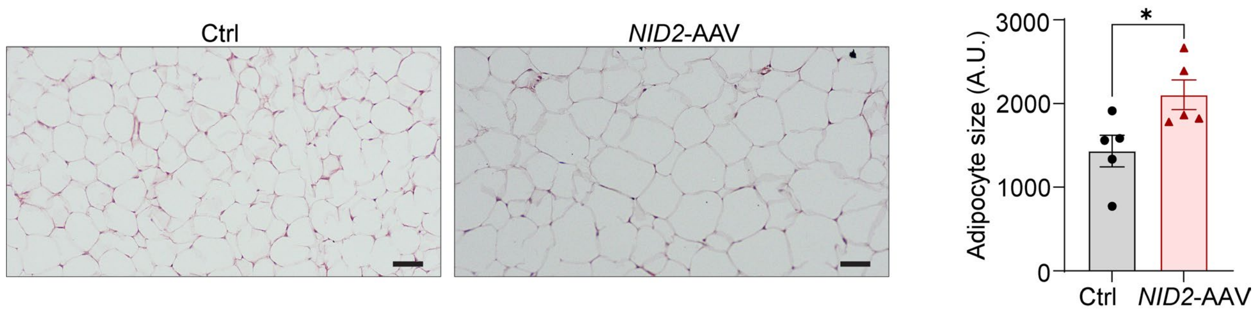

Figure S5: *NID2* overexpression in male mice increases adipocyte size. Representative images of EpiWAT sections stained with H & E, scale bar: 50 µm. The bar diagram represents mean adipocyte size in male control and *NID2*-AAV-injected *Apoe*$^{-/-}$ mice (n = 5). Statistical analyses were performed using a two-tailed unpaired t-test. Data represent mean ± SEM. $^{*}P < 0.05$.

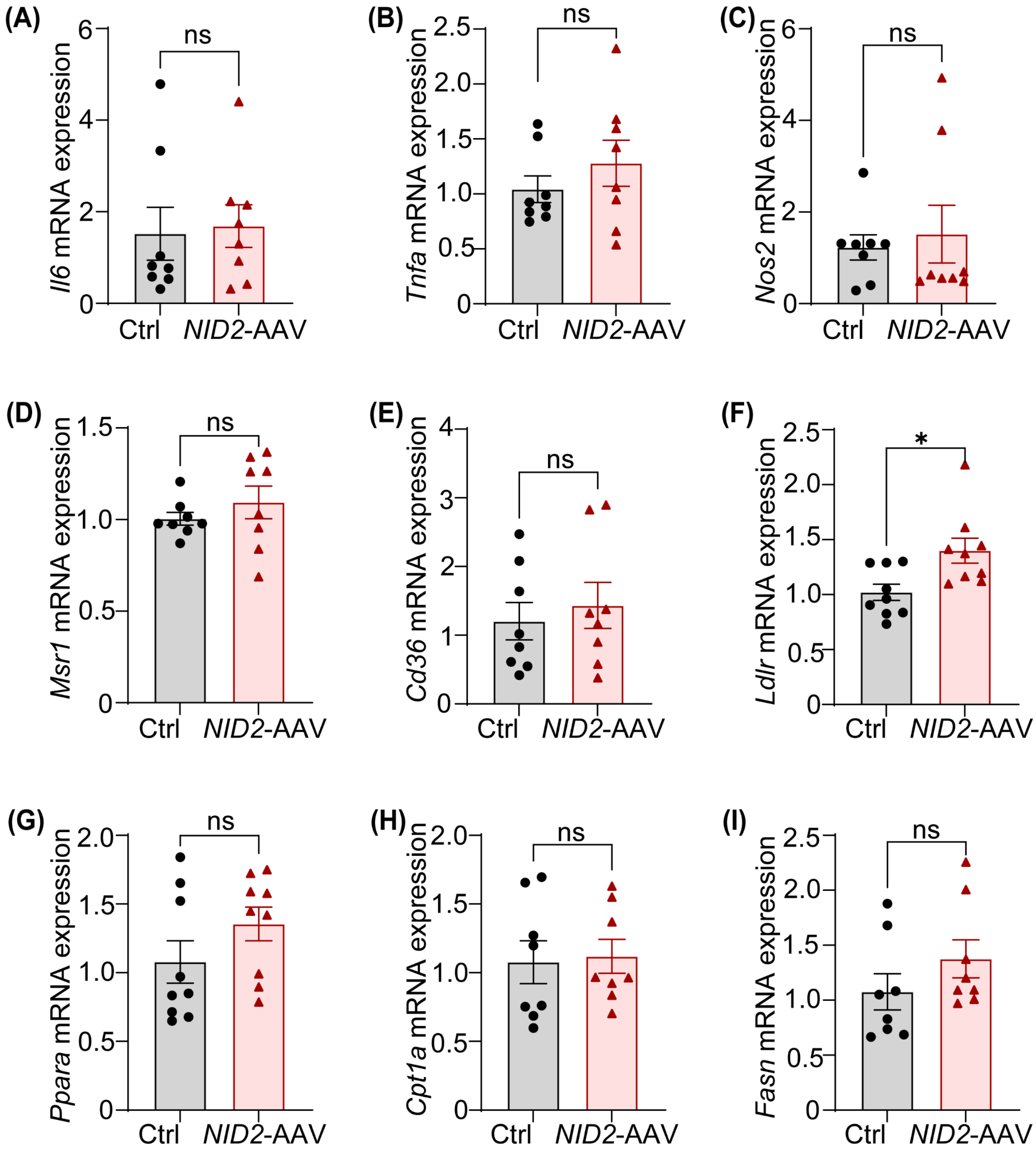

Figure S6: Expression of various lipid metabolism-related genes in the livers of control and *NID2*-overexpressing mice. (A - I) Bar diagrams represent relative mRNA expressions of various pro-inflammatory and lipid metabolism genes in the livers of control and *NID2*-AAV-injected mice using qRT-PCR, *Il6* **(A)**, *Tnfa* **(B)**, *Nos2* **(C)**, *Msr1* **(D)**, *Cd36* **(E)**, *Ldlr* **(F)**, *Ppara* **(G)**, *Cpt1a* **(H)** and *Fasn* **(I)**, (n = 8 - 9). Statistical analyses were performed using a two-tailed unpaired Mann-Whitney test **(A, C, E, F, and I)** and a two-tailed unpaired t-test, **(B, D, G and H)**. Data represent mean ± SEM. *$P < 0.05$.
